# Evaluation of the mineral composition, phytochemical and proximate constituents of three culinary spices in Nigeria: a comparative study

**DOI:** 10.1038/s41598-022-25204-3

**Published:** 2022-12-01

**Authors:** Uduenevwo Francis Evuen, Ngozi Paulinus Okolie, Augustine Apiamu

**Affiliations:** 1 Department of Biochemistry, College of Natural and Applied Sciences, Western Delta University, P.M.B. 10, Oghara, Delta State Nigeria; 2 Department of Biochemistry, Faculty of Life Sciences, University of Benin, P.M.B. 5025, Benin City, Edo State Nigeria; 3 Department of Biochemistry, Delta State University, P.M.B. 1, Abraka, Delta State Nigeria

**Keywords:** Biochemistry, Biological techniques, Plant sciences

## Abstract

Spices are prolific sources of phytochemicals of pharmaceutical and nutritional importance. They have been employed for centuries in the treatment of various maladies, in cuisines, and as inhibitors of oxidative degradation in foods. On this premise, a comparative assessment of the quantitative mineral composition, phytochemical and proximate constituents of *Xylopia aethiopica* (fruits)*, Piper guineense* (seeds), and *Rhaphiostylis beninensis* (roots) was done using standard protocols. Subsequently, methanol extracts of the spices were subjected to Gas Chromatography–Mass Spectrometry (GC–MS) analysis. Mineral analysis of the culinary spices revealed significant differences (*p* < 0.05) in the spices’ magnesium, zinc, iron, selenium, copper, calcium, manganese, molybdenum, potassium, and sodium contents. In the phytochemical analysis, flavonoids, phenols, and alkaloids (4.04%, 2.92%, 2.23%) predominate in *X. aethiopica*. Similarly, proximate analysis shows a preponderance of carbohydrates (81.24%) and proteins (4.83%) in *R. beninensis* and *P. guineense* respectively. However, values for the selenium (0.25 mg/L), saponin (0.23%), and moisture (0.71%) contents for *R. beninensis* were the lowest among the three spices. Results from the GC–MS analysis revealed the presence of thirteen, twelve, and thirteen phytoconstituents of *X. aethiopica, P. guineense*, and *R. beninensis* respectively. Prominent among them are hydrocarbons, acids, and esters with renowned biological attributes such as antioxidant, antimicrobial and anti-inflammatory. These findings indicate that the spices are notable wellsprings of bioactive components and justify their plethoric applications in Nigeria. Therefore, they could serve as lead compounds in the search for natural ingredients for drugs and nutraceuticals formulation.

## Introduction

Plants are known sources of a great category of bioactive chemical substances that function as biochemical and physiological agents in the body. Spices represent a class of plants with such effects. They are rich in aromatic compounds and have found wide applications in traditional medicine, industries, food preservation, and the improvement of sensory characteristics. Moreover, several ethnic cuisines are exceptionally certified owing to their spice constituents. A Few examples are Indian cuisine (turmeric), Thai cuisine (lemon grass, ginger, and, chili peppers), Italian cuisine (basil, sage, rosemary and oregano) and the African/Nigerian “Pepper soup” (bastered melegueta, clove, alligator pepper, ginger, black pepper, garlic, Ethiopian pepper, chilli peppers, and other spices)^1^.

A remarkable attribute of spices is their phytochemical constitution. The extraordinary benefits of phytochemicals have led researchers to continually unveil the additional usefulness of spices. Moreover, in recent times, there is an increase in the research on dietary minerals as a result of their importance in disease prevention coupled with the notable developments in the field of mineral research. *Xylopia aethiopica, Piper guineense,* and *Rhaphiostylis beninensis* are notable spices of culinary and ethnomedicinal importance in Nigeria.

*Xylopia aethiopica,* a deciduous tree that belongs to the plant family, *Annonaceae* is predominant in West Africa and is commonly referred to as *pepper tree*, *African guinea pepper, Ethiopian pepper,* or Senegal pepper^2^. In Nigeria, *X. aethiopica* has many vernacular names: *eeru* (Yoruba), *Kimba* (Hausa), *uda* (Igbo) and *urherien* (Urhobo). The medical importance of *X. aethiopica* has been reported^3^. *Raphiostylis beninensis* is a medicinal plant and a seasoning agent. The plant is called *atapata* (Yoruba), *osumadin* (Benin), *kpolokoto* (Igbo), *umeni* (Urhobo) and *kumeni* (Itsekiri)^4^. Some biological and pharmacological reports have also been made on the root bark extracts of *R. beninensis*^5,6^. *Piper guineense* is a West African spice plant commonly called Ashanti pepper. In Nigeria, it is known as *uziza* in Igbo and *Iyere* in Yoruba. It has other common names such as *Guinea pepper, Benin pepper, and False cubeb*^7^. *Piper guineense* is utilized in different forms for a variety of purposes; culinary, medicinal, cosmetic, and insecticidal uses^8^. In light of the general usefulness and importance of *Xylopia aethiopica, Piper guineense,* and *Rhaphiostylis beninensis*, the mineral composition, phytochemical, proximate and bioactive constituents of the culinary spices were evaluated for a broader application in foods and other relevant areas.

## Results

### Mineral composition of the spices

The mineral composition of *Xylopia aethiopica* (fruits), *Piper guineense* (seeds), and *Rhaphiostylis beninensis* (roots) are shown in Table 1. The sodium, potassium, magnesium, and manganese concentrations in *X. aethiopica* were significantly higher (*p* < 0.05) than those of *P. guineense* and *R. beninensis* spices*.* Moreover, *P.guineense* had significantly higher (*p* < 0.05) concentrations of calcium, molybdenum, and selenium mineral elements compared to the other two spices. Similarly, the iron, zinc, and copper concentrations in *Rhaphiostylis beninensis* were significantly higher (*p* < 0.05) than those of *Piper guineense* and *Xylopia aethiopica* spices*.* Generally, the highest and lowest concentrations of mineral elements in the three spices were found in iron and selenium.

**Table 1:** Mineral composition of selected spices.

Mineral elements	*X. aethiopica*	*P. guineense*	*R. beninensis*
Zn (mg/L)	4.09 ± 0.04^e^	1.11 ± 0.01^c^	7.33 ± 0.01^ k^
Ca (mg/L)	8.62 ± 0.02^p^	10.77 ± 0.01^j^	9.03 ± 0.01^ m^
Fe (mg/L)	14.07 ± 0.02^z^	11.16 ± 0.01^r^	16.03 ± 0.01f.
Se (mg/L)	0.45 ± 0.01^ g^	0.64 ± 0.02^b^	0.25 ± 0.01^q^
Na (mg/L)	6.08 ± 0.01^d^	4.98 ± 0.01^m^	3.72 ± 0.01^c^
Mo (mg/L)	1.09 ± 0.01^x^	3.07 ± 0.01^v^	2.33 ± 0.01^t^
Mg (mg/L)	7.54 ± 0.01^ s^	4.38 ± 0.04^k^	5.95 ± 0.02^y^
Cu (mg/L)	3.95 ± 0.01^j^	5.58 ± 0.01^a^	6.82 ± 0.02^z^
Mn (mg/L)	5.47 ± 0.01^u^	4.75 ± 0.01^f^	2.43 ± 0.01^ h^
K (mg/L)	11.31 ± 0.02^c^	8.81 ± 0.01^s^	6.55 ± 0.01^j^

Values are expressed as mean ± standard error of mean (*X* ± S.E.M.) in triplicate. Values with different letter along the same row are significantly different (*p* < 0.05).

### Phytochemical Constituents of the Spices

Table 2 below reveals the quantitative phytochemical constituents of *R*. *beninensis, P. guineense* and *X. aethiopica* spices. The flavonoid, alkaloid, and phenol contents of *X. aethiopica* were significantly higher (*p* < 0.05) than those of *P. guineense* and *R. beninensis* spices respectively. The tannin content of *R*. *beninensis* was significantly higher (*p* < 0.05) than those of *P. guineense* and *X. aethiopica* spices. However, there were no significant differences (*p* > 0.05) in the tannin content of *P. guineense* and *X. aethiopica* spices respectively. A similar trend was observed in the Oxalate contents of *R. beninensis* and *X. aethiopica* spices and the Phytate contents of *R*. *beninensis* and *P. guineense* spices respectively. In the same vein, no significant differences (*p* > 0.05) were observed in the saponin contents of the three spices.

**Table 2:** Phytochemical constituents of selected spices.

Phytochemicals	*R. beninensis*	*P. guineense*	*X. aethiopica*
Flavonoids (%)	^*¶*^3.72 ± 0.13^a^	2.73 ± 0.08^b^	4.04 ± 0.09^c^
Tannins (%)	^*¶*^0.78 ± 0.04^a^	0.22 ± 0.02^b^	0.17 ± 0.02^b^
Alkaloids (%)	^*¶*^1.74 ± 0.07^b^	1.57 ± 0.03^b^	2.23 ± 0.05^c^
Phenols (%)	^*¶*^2.03 ± 0.07^a^	0.33 ± 0.02^b^	2.92 ± 0.16^c^
Saponins (%)	^*¶*^0.23 ± 0.01^b^	0.36 ± 0.06^b^	0.28 ± 0.01^b^
Phytate (%)	0.57 ± 0.02^a^	0.66 ± 0.02^a^	0.42 ± 0.02^b^
Oxalate (%)	0.31 ± 0.02^b^	0.05 ± 0.01^a^	0.25 ± 0.04^b^

Values are expressed as mean ± standard error of mean (*X* ± S.E.M.) in triplicate. Values with different letters along the same row are significantly different (*p* < 0.05).

^¶^Values derived from our previous published work^6^.

### Proximate composition of the spices

The proximate composition of dried fruits of *X. aethiopica*, dried seeds of *P. guineense,* and dried roots of *R. beninensis* are shown in Table 3*.*

**Table 3:** Proximate composition of *R. beninensis, P. guineense,* and *X. aethiopica* spices.

Parameters	*R. beninensis*	*P. guineense*	*X. aethiopica*
Moisture content (%)	0.71 ± 0.01^a^	0.82 ± 0.01^b^	1.13 ± 0.02^c^
Crude protein (%)	3.82 ± 0.08^a^	4.83 ± 0.09^b^	3.14 ± 0.05^c^
Lipid (%)	0.39 ± 0.01^a^	1.84 ± 0.01^b^	13.82 ± 0.04^c^
Ash (%)	7.43 ± 0.07^a^	6.22 ± 0.08^b^	6.47 ± 0.08^b^
Crude Fibre (%)	6.42 ± 0.01^b^	6.35 ± 0.04^b^	5.36 ± 0.05^a^
Carbohydrate (%)	81.24 ± 0.25^b^	79.93 ± 0.11^b^	70.08 ± 0.30^a^

Values are expressed as mean ± standard error of mean (*X* ± S.E.M.) in triplicate. Values with different letters along the same row are significantly different (*p* < 0.05).

The moisture, protein, and lipid contents of the 3 spices were significantly different (*p* < 0.05) from each other. Moreover, *X. aethiopica* had the highest moisture and lipid contents while *P. guineense* and *R. beninensis* had the highest protein and carbohydrate contents respectively. However, there were no significant differences (*p* > 0.05) in the fibre and carbohydrate contents of *R*. *beninensis* and *P. guineense* spices respectively. A similar trend was also observed in the ash contents for *P. guineense* and *X. aethiopica* spices respectively.

### Bioactive compounds identified in the spices by GC–MS analysis

**Figure 1:**
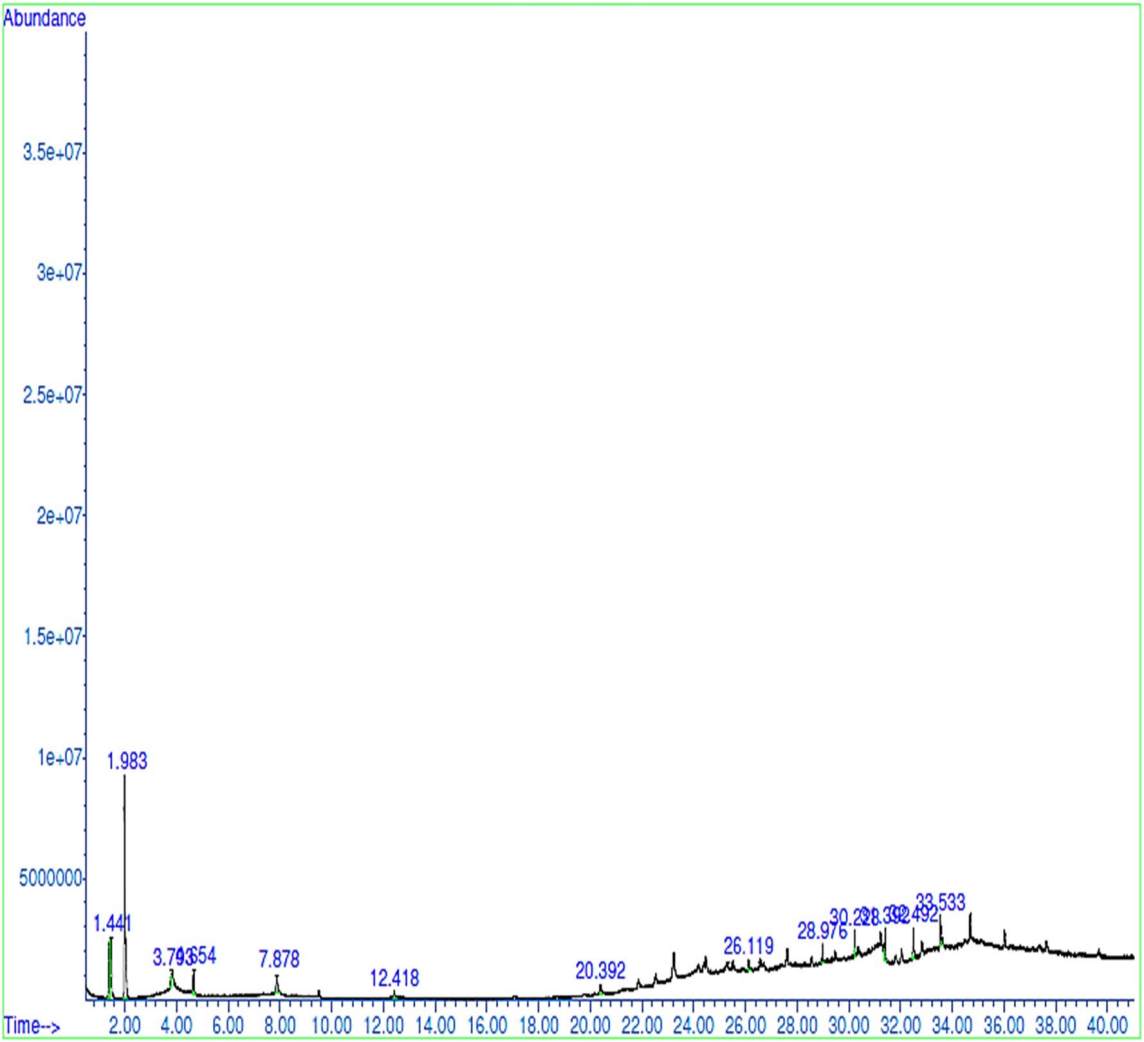
GC–MS Chromatogram of *X. aethiopica* Fruits.

**Figure 2:**
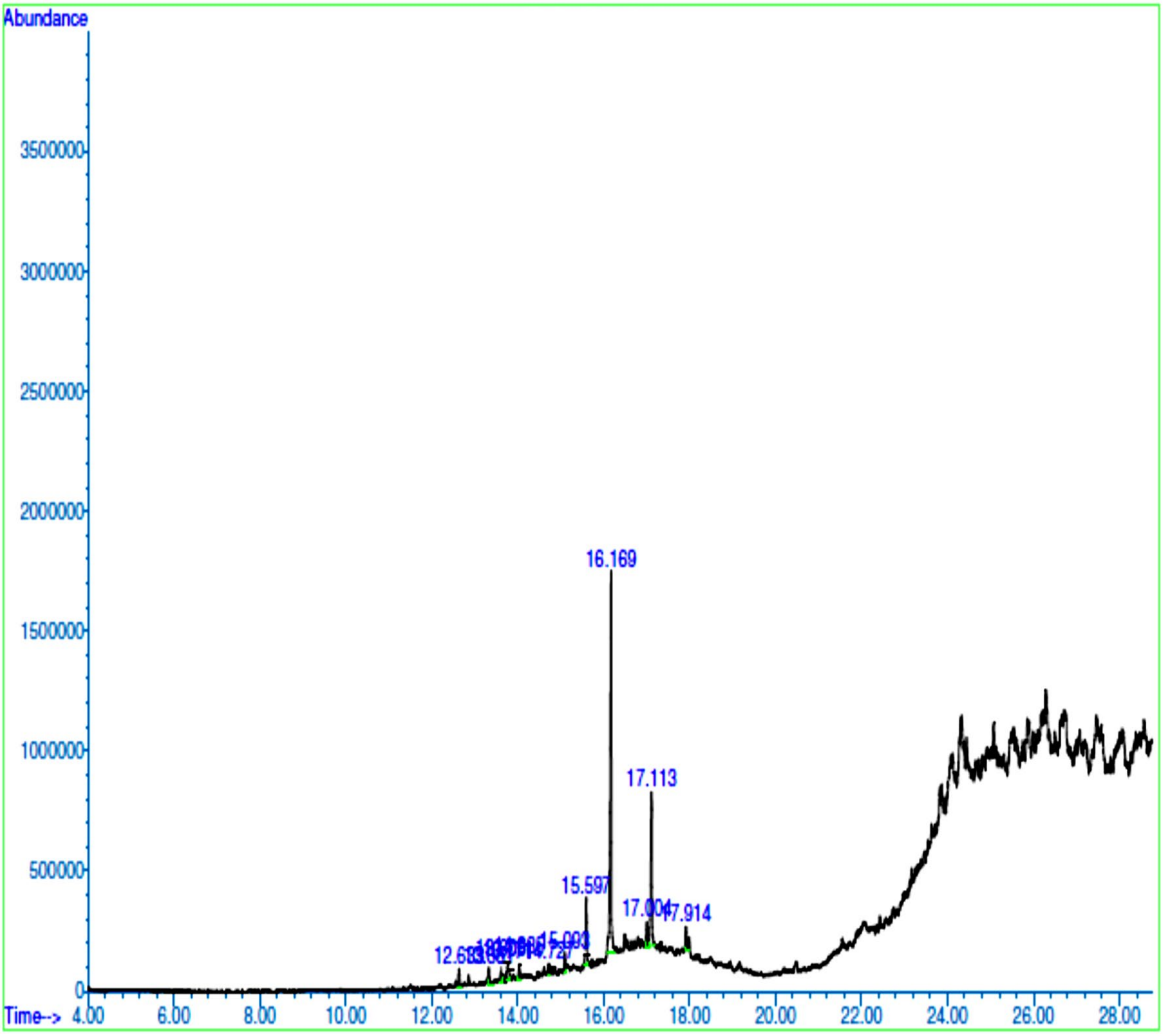
GC–MS chromatogram of *Piper guineense* seed extract.

**Figure 3:**
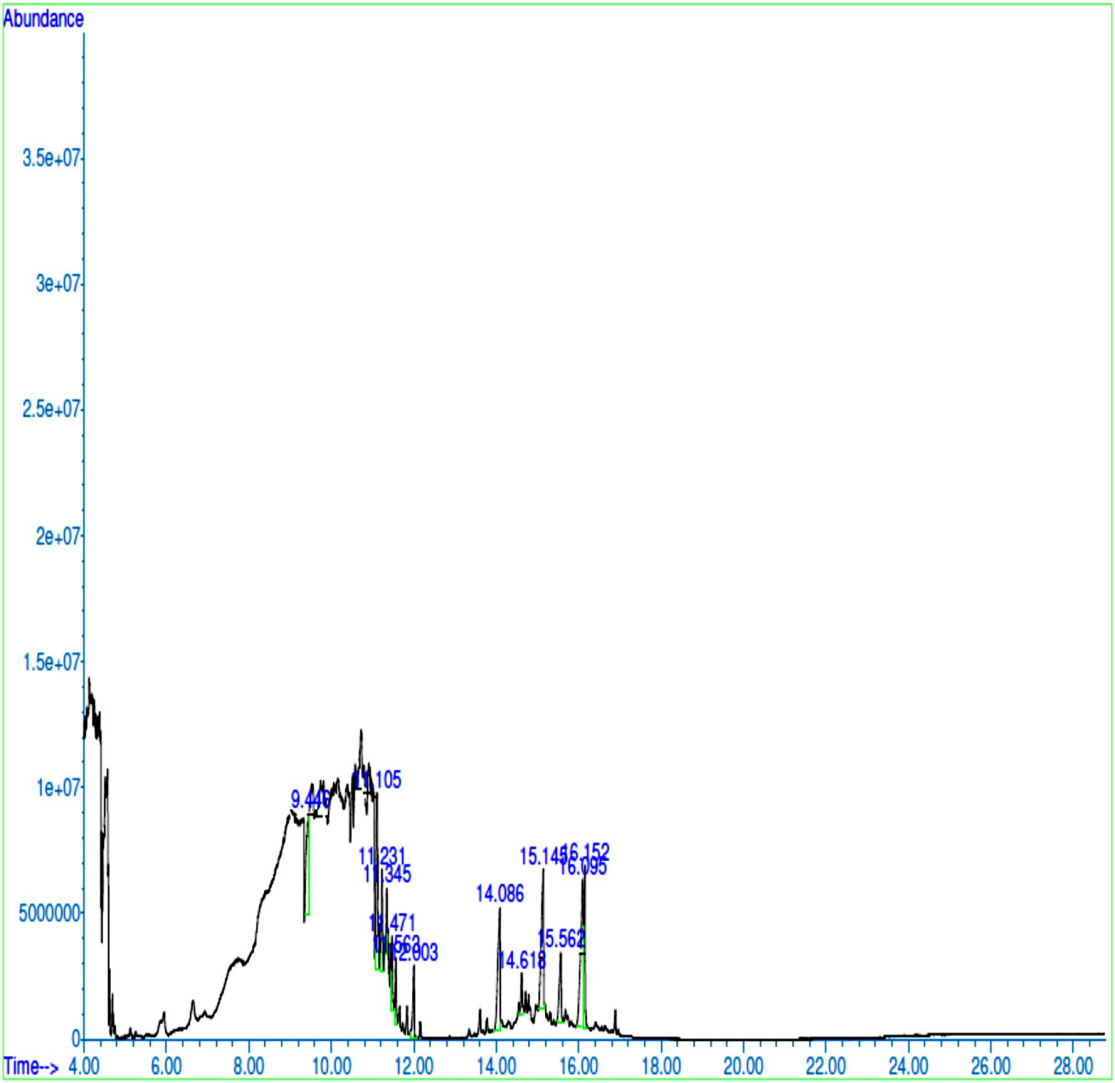
GC–MS chromatogram of *Rhaphiostylis beninensis* root extract.

The GC–MS chromatograms of methanol extracts of *X. aethiopica* fruits, *P. guineense seeds and, R. beninensis* roots displayed thirteen, twelve, and thirteen major peaks respectively representing their phytocomponents (Figs. 1, 2, and 3). The identities of the various phytocomponents in the extracts of *Xylopia aethiopica* fruits, *Piper guineense* seeds and *Rhaphiostylis beninensis* roots and their reported biological properties are highlighted in Tables 4, 5, and 6 respectively. Generally, major bioactive compounds in each of the spices such as Catechin (39.54%), 4H-1-Benzopyran-4-one, 5-hydroxy-7-methoxy-2-methyl-(50.18%), and 4H-1-Benzopyran-4-one, 7-hydroxy-3-(4-methoxyphenyl)- (16.27%) are phenolic compounds.

**Table 4:** Bioactive compounds identified in methanol extracts of *X. aethiopica* fruits.

Peaks	Compound	Relative abundance (%)	Molecular formula	Retention time (Mins.)	Molecular weight (g/mol)	Structure	Biological activity
1	Methanesulfinothioic acid, S-1-propyl ester	21.297	C_4_H_10_OS_2_	1.441	138.02		Antimicrobial^9^
2	Catechin	39.538	C_15_H_14_O_6_	1.983	290.08		Antioxidant^10^, Antibacterial [^11^, Antifungal^12^, Hepatoprotective^13^
3.	2-Thiophenecarboxaldehyde,4-(1H-1,3benzimidazolel-1-ylmethyl)-5-methyl-	3.138	C_14_H_12_N_2_OS	3.793	256.07		Antioxidant^14,15^,
4	Daidzein, Bis (heptafluorobutyrate)	5.708	C_23_H_8_F_14_O_6_	4.654	646.01		Antioxidant^16^
5	2-Amino-3-(4-hydroxyphenyl)-propanoic acid	5.407	C_9_H_11_NO_3_	7.878	181.07		Antimicrobial^17^
6	Glycyl-L-tyrosine	1.617	C_11_H_14_N_2_O_4_	12.418	238.10		Growth promoter, Nitrogen balance^18^
7	2-n-Hexylphenol	2.694	C_12_H_18_O	20.392	178.16		–
8	3-Hexadecylaminopyridine	1.588	C_21_H_38_N_2_	26.119	318.30		–
9	α-Methyltyrosine, N,O-diacetyl-	2.693	C_14_H_17_NO_5_	28.976	279.11		–
10	4′-Methoxy-5,7-dihydroxy isoflavone	3.078	C_16_H_12_O_5_	31.392	284.07		Antioxidant^19^
11	Coumaran-5-ol-3-one, 2-[4-hydroxy-3-methoxybenzylidene]-	4.501	C_16_H_12_O_5_	32.492	284.07		Antimalarial^20^, Anti-histamine^21^
12	1(2H)-Naphthalenone, 3,4-dihydro-2-(1-naphthalenylmethylene)-	4.592	C_21_H_16_O	33.533	284.12		–
13	9-(o-Toluidino)acridine	4.151	C_20_H_16_N_2_		284.13		Antiviral^22^, Antibacterial^23^ Anticancer^24^

**Table 5:** Bioactive compounds identified in *P. guineense* seeds.

Peak	Compound	Relative Abundance (%)	Molecular Formula	Retention Time (Mins.)	Molecular Weight (g/mol)	Structure	Biological activity
1	2-Pentanone,4-cyclohexylidene -3,3-diethyl	2.390	C_15_H_26_O	12.633	222.20		Antivenom^25^
2	Benzoic acid, 4-hydroxy-	2.373	C_7_H_6_O_3_	13.331	138.03		Antimicrobial^26^
3	Trans-Ferulic acid	2.121	C_10_H_10_O_4_	13.605	194.16		Antioxidant, Antibacterial, Anti-inflammatory UV absorptive^27,28^
4	Propenoic acid, 3-(1-ethyl-3,5-dimethyl-4-pyrazolyl)-	3.100	C_10_H_14_N_2_O_2_	13.771	194.11		–
5	Thiazolidin-4-one,5-(2,5-dimethoxybenzylidene)-3-pyridin-3-ylmethyl-2-thioxo-	1.985	C_18_H_16_N_2_O_3_S_2_	14.035	372.06		Antioxidant, Antitumor^29^
6	5,6,7,4′-Tetramethoxy Flavanone	1.810	C_19_H_20_O_6_	14.727	344.13		Anticancer^30,31^
7	Taxifolin	2.014	C_15_H_12_O_7_	15.093	304.06		Antioxidant^32,33^
8	Taxifolin	7.922	C_15_H_12_O_7_	15.597	304.06		Antioxidant^32,33^
9	4H-1-Benzopyran-4-one, 5-hydroxy- 7-methoxy- 2-methyl-	50.177	C_11_H_10_O_4_	16.169	206.06		Antimicrobial^34^
10	Lathodoratin	2.601	C_11_H_10_O_4_	17.004	206.06		Antitumor^35^ Anti-inflammatory^36^ Antispasmolytic^37^
11	Quinoline, 2-(2-pyridinyl)-	20.202	C_14_H_10_N_2_	17.113	206.04		Antimalarial, Anticancer, Antihelmintic ^38^
12	Hesperetin	3.304	C_16_H_14_O_6_	17.914	302.08		Antioxidant, Anti-inflammatory^39,40^

**Table 6:** Bioactive compounds identified in *R. beninensis* roots.

Peaks	Compound	Relative Abundance (%)	Molecular Formula	Retention Time (Mins.)	Molecular Weight (g/mol)	Structure	Biological activity
1	Bicyclo[3.1.0]hexane- 6-methanol, 2-hydroxy-1,4,4-trimethyl-	11.02	C_10_H_18_O_2_	9.446	170.13		Anti-Candida, Anti-inflammatory^41,42^
2	4-Terpinenyl acetate	13.26	C_12_H_20_O_2_	11.105	196.15		Antioxidant Antimicrobial^43^
3	Quercetagetin	4.92	C_15_H_10_O_8_	11.231	318.04		Antioxidant Antilipemic Antidiabetic^44^
4	Methanone, (3-benzoyl-2,6-dihydroxyphenyl)phenyl-	2.31	C_20_H_14_O_4_	11.345	318.09		–
5	Bis(trimethylsilyl) 4-methoxyphenylphosphonate	2.39	C_13_H_25_O_4_PSi_2_	11.471	332.10		–
6	L-Tyrosine, N-(trifluoroacetyl)-, trimethylsilyl ester, trifluoroacetate (ester)	2.93	C_16_H_17_F_6_NO_5_Si	11.563	445.08		–
7	Benzoic acid, 3,4,5-trihydroxy-	4.32	C_7_H_6_O_5_	12.003	170.02		Antimicrobial^45^ Analgesic^46^ Anti-HIV^47^
8	Apigenin	11.94	C_15_H_10_O_5_	14.086	270.05		Antipyretic Antiviral Antioxidant^48,49^ Antimicrobial^50^
9	4′-Methoxy-5,7-dihydroxy isoflavone	2.54	C_16_H_12_O_5_	14.618	284.07		Estrogenic Anti-inflammatory Anti-proliferative Antioxidant ^51^
10	Genkwanin	13.73	C_16_H_12_O_5_	15.145	284.07		Anti-inflammatory^52^ Antimicrobial^53^ Anti-plasmodial^54^ Anti-radical^55^
11	9,10-Anthracenedione, 1,8-dihydroxy-4-methoxy- 2-methyl-	5.73	C_16_H_12_O_5_	15.562	284.07		–
12	4H-1-Benzopyran-4-one, 7-hydroxy-3-(4-methoxyphenyl)-	16.27	C_16_H_12_O_4_	16.095	268.07		Anticancer^56,57^
13	Thioflavin t	8.66	C_17_H_19_ClN_2_S	16.152	318.10		Antioxidant Anti-Inflammatory Anti-Obesity^58^

## Discussion

### Mineral composition of the spices

Spices are proven sources of vital nutrients necessary for the growth and sustenance of various physiological processes of the body hence, lack of an adequate quantity of these nutrients may lead to a host of diseased conditions. In the present study, Iron which is an essential trace element for the synthesis of haemoglobin, and normal functioning of the central nervous system, was the most abundant mineral in all the three spices evaluated. It ranged from 11.16 to 16.03 mg/L with *Rhaphiostylis beninensis* having the highest amount and *Piper guineense* having the lowest amount. Moreover, the considerable amount of copper (6.82 mg/L) present in *Rhaphiostylis beninensis* could have actuated the release of iron in the formation of haemoglobin. Hence, the consumption of foods or supplements prepared with *Rhaphiostylis beninensis* roots may supply more iron to the body necessary for oxygen transport in the haemoglobin of erythrocytes. Lasisi et al*.*^59^ reported that the spice is utilized as a tonic for children between the ages of two to three years and for the treatment of a diseased condition that makes the whole skin turn white (*afun)* in the South-Western region of Nigeria. Similarly, in *X. aethiopica and P. guineense* spices, the relatively high proportions of Iron have given a better understanding of their applications in the preparation of the renowned “pepper soup” for women immediately after delivery in several parts of Nigeria^60^. Thus, these studies affirm the haematinic attribute of the spices. Manganese which is a known activator of several enzymes and also necessary for the formation of haemoglobin predominates in *Xylopia aethiopica*. This outcome may have contributed to the spice’s haematinic property.

Zinc has been reported to exhibit catalytic and modulatory activities on over 300 enzymes. It also aids in the maintenance of a healthy immune system and enhances sperm development, ovulation and fertilization^61^. The significantly higher (*p* < 0.05) concentration of Zinc observed in *Rhaphiostylis beninensis* than in the other two spices could be traceable to its reported pro-sexual attributes^62^.

Zinc acts as a vital component in male and female reproductive prospects. It cannot be stored in the human body. Consequently, the consumption of zinc in diets is the only means of sustaining the body’s physiological activities, particularly in males and females who have attained the age of reproduction. Therefore, diets supplemented with *Rhaphiostylis beninensis* may serve a better chance of enhancing the reproductive potentials of men and women forgoing treatment for infertility than those with *X. aethiopica and P. guineense* spices.

Sodium and potassium present in relatively high concentrations in *X. aethiopica* are major cations present in extracellular and intracellular fluids respectively. They assist in sustaining electrolyte balance in body fluids. The higher significant concentration (*p* < 0.05) of sodium is an indication that the spice will possess the capacity to assist in osmotic balance regulation and maintenance of the body’s internal environment in comparison with the other two spices. In the same vein, the higher significant level (*p* < 0.05) of potassium in the said spice shows that; it will act in synergy with sodium to enhance the above functions. A previous similar report ^63^ has also revealed relatively higher concentrations of potassium (277.34 mg/100 g) in *Xylopia aethiopica* compared to those of other culinary spices such as *Momodara myristica, Allium cepa, Zingiber officinale, Ocimum gratissium* evaluated in course of their study. Consequently, the consumption of food substances containing *X. aethiopica* may aid in the prevention of diseased conditions linked with sodium and potassium deficiencies.

Magnesium is essential in glucose and insulin metabolism chiefly by enhancing tyrosine kinase activity of the insulin receptor. The activity of phosphorylase b kinase is also activated by magnesium thereby bringing about the release of glucose-1-phosphate from glycogen^64^. Thus, it could be deduced that *Xylopia aethiopica* may be a better candidate for the formulation of chemotherapeutic agents for diabetic conditions associated with dysfunctional insulin than *Piper guineense* and *Rhaphiostylis beninensis*.

*Piper guineense* contains the highest concentration of calcium (10.77 mg/L) of the three spices. A previous similar study^65^ also reported that the concentration of calcium (146.43 mg/Kg) was the highest of all minerals present in this spice. The said concentration was also higher than that of *X. aethiopica* (98.40 mg/Kg) in accordance with the findings of this study. This indicates that the seeds of the spice may play vital roles in good teeth and bone development coupled with its essential role as a cofactor in various enzyme-catalyzed reactions such as blood clotting and several other physiological processes. Plausibly, *Piper guineense* seeds may be employed in the management of bone-related disorders associated with calcium deficiency such as osteoporosis in postmenopausal women.

The relative concentrations of molybdenum and selenium in the spices were low compared with those of other elements. Though, present in a meagre portion of the spices, they contribute to the total well-being of the human body. Molybdenum assists in the inhibition of pulmonary and liver fibrosis. Furthermore, enzymes involved in energy metabolism are also activated by molybdenum. Selenium, on the other hand, is vital for a robust immune system, production of “good” prostaglandins, and fertility ^66^.

To the best of our knowledge, this is the first report on the mineral composition of root extracts of *R. beninensis.* However, values reported for the levels of iron (2.41 mg/L, 2.73 mg/Kg, 2.65 mg/Kg), sodium (4.03 mg/L), copper (0.08 mg/L, 0.41 mg/Kg, 0.01 mg/Kg), Zinc (0.42 mg/L, 0.37 mg/Kg, 0.31 mg/Kg), and manganese (0.32 mg/L, 2.06 mg/Kg, 0.19 mg/Kg) for *X.aethiopica* and *P.guineense* from previous similar studies^60,65^ were lower than the values obtained in this study. The discrepancies observed in values could be attributed to differences in methods employed during analysis, stage of maturity of the fruits/seeds before harvesting them, nature of the soil, and climatic factors of the geographical region where the spices were harvested. Contrarily, values of 8.81 mg/L and 10.77 mg/L obtained for potassium and calcium levels in *P. guineense* in this study are comparable to 8.87 ppm and 11.20 ppm obtained by Imo et al.^60^.

### Phytochemical constituents of the spices

Phytochemical evaluation of the dried roots of *Rhaphiostylis beninensis*, dried seeds of *Piper guineense* and dried fruits of *Xylopia aethiopica* revealed the presence of flavonoids, alkaloids, phenols, saponins, Phytate, Oxalate, and tannins in varying concentrations (Table 2). The presence of the above phytochemicals in *Xylopia aethiopica* aligns with earlier reports^67,68^. However, the relative compositions of alkaloids (2.23%), flavonoids (4.04%), and saponins (0.28%) in the fruit extracts of *X. aethiopica* were higher than those of Uhegbu et al*.*^69^: alkaloids (1.49%), flavonoids (0.22%) and saponins (0.18%). The observed differences may be due to the method of analysis, harvesting time, climatic conditions of the growing area, and variation in solvent for extraction.

The phytochemical results obtained for the root of *R. beninensis* are in agreement with previous studies by Ofeimum and Mbionwu^70^ in which the methanol root extract of the plant gave a higher concentration of flavonoids compared to its alkaloid and tannin contents respectively. Similarly, findings on the phytochemical components of *P. guineense* are in line with the reports of previous authors^71,72^. Echo et al*.*^71^ also reported that the phytochemical composition of alkaloids in *P. guineense* was 1.67% which was comparable to 1.57% obtained in this study. This study also observed that the percentage composition of tannins is 0.22% in seeds of *P. guineense* which was also comparable to the 0.30% reported by Omodamiro and Ekeleme^72^.

Okwu^73^ reported that the mean percentage alkaloid and saponin contents of *P. guineense* seeds were 1.20% and 0.45% respectively which were comparable to 1.57% and 0.36% respectively obtained for *P. guineense* seeds in this study. Qiu^74^ have shown that alkaloids have a wide range of pharmacological activities. Hence, the presence of alkaloids in *X. aethiopica, R. beninensis and P. guineense* spices could account for their use as antimicrobial agents.

A growing interest exists in the Flavonoids and phenol contents of plants owing to their roles against pathogenic organisms and in the scavenging of free radicals. Flavonoids were found to be the most abundant phytochemical in all the spices; *X. aethiopica* (4.04%), *Piper guineense* (2.73%), and *R. beninensis* (3.72%). Flavonoids and phenols are known antioxidants in plants and humans. Hence, *X. aethiopica* may have a greater antioxidant potential in comparison with the other two spices owing to its higher constituent of flavonoids and phenols.

Tannins are aromatic compounds containing phenolic groups. They are one of the principal active ingredients found in plant-based medicines possessing antiviral, antibacterial, and antitumor activities. Tannins significantly predominate (*p* < 0.05) in *R. beninensis.* Consequently, *R. beninensis* may serve a better potential as a major active ingredient in drug production compared to the other two spices^75,76^.

Oxalates and phytates possess potent binding affinities to vital minerals such as calcium, iron, and zinc at high concentrations. Thus, they may be regarded as anti-nutritional factors^77,78^. The phytate and Oxalate compositions of the samples analyzed ranged from 0.42 to 0.57% and 0.03% to 0.31% respectively. Plausibly, the above amounts may not pose any health hazard.

Roa et al.^79^ have shown that saponins possess antioxidant, antitumor, and anti-mutagenic activities and may also reduce the incidence of human cancers by inhibiting the growth of cancer cells. The saponin content of the spices ranged from 0.23 to 0.28%. Interestingly, toxicological studies of saponin using relevant experimental models have established that even at a higher concentration of 3.5%, saponin was safe and did not cause any systemic side effects^80^. Thus, it can be deduced from the above that the levels of saponin in the three spices are safe for human consumption.

### Proximate composition of the spices

Findings on the nutritional components of the three spices, *Rhaphiostylis beninensis*, *Piper guineense,* and *Xylopia aethiopica* are shown in Table 3.

*X. aethiopica* and *R. beninensis* had the highest and lowest percentage moisture contents respectively of the three spices. The proximate data obtained for the moisture contents of *Piper guineense* and *Xylopia aethiopica* spices reported in this work does not agree with those of Borquaye et al*.*^65^ who reported higher moisture content values for the spices. The observed difference in values may be due to differences like the soil and climatic conditions in the areas of cultivation, genetic variations, and differences in analytical procedures.

The values obtained for the percentage moisture contents of the three spices range from 0.71% to 1.13%. These values indicate that the spices are relatively dry owing to their low moisture content. Moreover, moisture was the lowest amount among all proximate parameters evaluated in the three spices. Low moisture content prevents quick deterioration of food materials and deters the activities of food spoilage microorganisms. Consequently, the three spices in this study can be stored for a longer period.

The ash content obtained for the three spices under this study ranged from 6.22 to 7.43%. *Raphiostylis beninensis* had the highest value while *P. guineense* had the lowest value. Result obtained for the ash content of *P. guineense,* 6.22% is in line with the reports of Negbenebor et al*.*^81^ whose value obtained was 6.33%. Ash content connotes the mineral composition of the spices. These minerals are essential for the proper functioning of the human immune system. There were no significant differences (*p* > 0.05) in the ash contents of *P. guineense* and *X. aethiopica* spices. Therefore, both spices may have a similar and lower composition of vital mineral elements compared to *R. beninensis* spice.

The crude protein content of the spices is in the range of 3.14–4.83% with *P. guineense* seeds having the highest and *X. aethiopica* having the lowest protein contents respectively. The percentage mean crude protein content, 4.83% obtained in this study is comparable to 5.86% and 5.57% obtained by Negbenebor et al*.* and Uhegbu et al*.*^69,81^ respectively for *P. guineense* seeds. However, the percentage mean crude protein content obtained for *X. aethiopica,* 3.14% in this study was lower than 7.73% and 11.90%obtained by Borquaye et al*.* and Uhegbu et al*.*^65,69^ respectively in a similar study.

The observed differences in crude protein content obtained for *X. aethiopica* fruits could have resulted from variations in the solvents for the extraction or analytical procedure. Notwithstanding, the proteins present in the three spices could impact the proteins required by humans for certain biochemical activities or processes such as replacement and repair of worn-out tissues, growth, provision of hormones, and amino acids. Hence, crude protein values obtained for spices in this study make them good sources of plant protein.

Fibre content was highest in *R. beninensis* (6.42%), followed by *P.guineense* (6.35%) and subsequently, *X. aethiopica* (5.36%). There were no significant differences (*p* > 0.05) between the fibre contents of *R. beninensis* and *P.guineense* spices. Thus, both spices could serve as a good source of fibre in the diet compared with *X. aethiopica*. Moreover, adequate intake of dietary fibre could aid absorption of water from the body, bulky stool, digestion, and the prevention of constipation. Interestingly, this is the first time, data on the proximate composition of *R. beninensis* spice is presented in Literature to the best of our knowledge. However, values obtained for the fibre content of *P.guineense* seeds are comparable to that of a similar study conducted by Negbenebor et al*.*^81^. In that work, the mean percentage crude fibre content of *P.guineense* seeds was estimated as 8.79% while that of this study is 6.35%. In the same vein, the values obtained by Okwu^73^ and Okwu and Josiah^82^ for *P.guineense* seeds (4.31%) and *X. aethiopica* fruits (6.44%) were also comparable to the 6.35% and 5.36% obtained respectively for the said spices. The lipid content of the spices were in the range of 0.39–13.82% with *R. beninensis* and *X. aethiopica* having the lowest and highest amounts respectively. Lipids are excellent sources of energy. They also aid in the transport of fat-soluble vitamins. The low amount of lipid obtained for *R. beninensis* (0.39%) and *P. guineense* (1.84%) spices respectively, implies that they can be recommended as part of a weight loss regimen. However, *X. aethiopica* may support the production of hormones of lipid origin owing to its higher amount of lipids.

In the same vein, Uhegbu et al*.*^69^ obtained 10.64% as the percentage lipid content for *X. aethiopica* fruits. This value is lower than a value of 13.82% obtained in this study. However, a value of 6.73% obtained by Imo et al*.*^60^ for *X. aethiopica* fruits does not agree with the 13.82% obtained in this study. This may be a result of differences in the solvent used for extraction or environmental factors.

Carbohydrate content had the highest nutritional composition of all the spices evaluated in this study. It ranged from 70.08 to 81.24% with *X. aethiopica* having the lowest amount and *R. beninensis* having the highest amount. Carbohydrates such as glucose provide energy to cells in the body, especially the brain, which solely depends on glucose for energy. Therefore, the high carbohydrate contents observed for the three spices indicate that they are good sources of fuel and energy for the body’s daily activities. Effiong et al*.*^83^ obtained 69.46% as the mean percentage content of carbohydrates in *X. aethiopica*. The value obtained by the said authors is in consonance with 70.08% obtained in this study. However, a lower value of 26.08% recorded by Imo et al*.*^60^ was not in line with the value obtained in this study. For *P. guineense,* results from earlier studies^65,73^ estimated the percentage carbohydrate content of the spice as 48.77% and 40.29% respectively. The values reported were lower than a value of 79.93% obtained in this study. This disparity in results could be a consequence of variations in environmental conditions during the cultivation of the spices or methods of analysis.

### Bioactive compounds identified in the spices by GC–MS analysis

Polyphenolic compounds which constitute a major proportion of the bioactive components of each of the spices are well known for their numerous biological properties such as antioxidant, antimicrobial, and anti-inflammatory^84,85^. A Previous similar study in which active principles were identified in *X. aethiopica,* also revealed the presence of potent phenolic compounds such as apigenin, caffeic acid, chlorogenic acid, ellagic acid, kaempferol, rutin and quercetin ^86^. In the same vein, Adefegha et al.^87^ detected quercetin and isoquercitrin in *P. guineense* during Chromatographic profiling of its seeds. However, no previous reports were available on the GC–MS fingerprints for *R. beninensis* to the best of our knowledge.

In Nigeria, the therapeutic application of these spices in folklore medicine could be attributed to their bioactive constituents. For example, the use of *X. aethiopica* for the treatment of malaria, diarrhoea and infections in rural areas^88^ may be traced to the reported biological activities of Methanesulfinothioic acid, S-1-propyl ester, Catechin, 2-Thiophenecarboxaldehyde, 4-(1H-1,3-benzimidazol-5-methyl-, 2-Amino-3-(4-hydroxyphenyl)-propanoic acid, Coumaran-5-ol-3-one, 2-[4-hydroxy-3-methoxybenzylidene]-, 9-(o-Toluidino) acridine present in the spice.

Methanesulfinothioic acid, S-1-propyl ester; a thiosulfinate has been reported to possess antimicrobial activity^9^. Thiosulfinates are unstable volatile organosulphur compounds known for imparting characteristic aroma and taste to plants. They have also been identified as one of the bioactive components in the culinary plant, Onion (*Allium cepa*)^9^. This plant is an essential component of several ethnic cuisines^1^. In addition, 2-Amino-3-(4-hydroxyl)-propanoic acid which was also among the bioactive compounds isolated from *Astropecten spinulosus,* exhibited antimicrobial properties^17^. Moreover, amino acridines such as 9-(o-Toluidino) acridine, identified as part of the GC–MS fingerprints of the spice, exhibited bioactivities such as antiviral^22^, antibacterial^23^ and anticancer^24^. Thus, the 9-(o-Toluidino) acridine may serve as a lead molecule in the synthesis of various chemotherapeutic agents. The bioactive compound, 2-Thiophenecarboxaldehyde, 4-(1H-1,3-benzimidazolbenzimidazole-5-methyl- is a derivative of Benzimidazole. The derivatives of Benzimidazole have been reported to exhibit antioxidant properties^14^. More so, Archie et al*.*^15^ have also shown that 2-substituted benzimidazoles demonstrated antioxidant abilities. Moreover, the structures of some antibacterial and antifungal drugs of clinical importance today such as cimetidine, omeprazole, and, flumazenil, have imidazole rings serving as a pharmacophoric moiety or substituent^89^. Antimicrobials have also been reported to be relatively safe and useful in the extension of the shelf life of foods, hence, they render food safe for consumption^90^.

Catechins exhibit numerous health benefits by scavenging free radicals, inhibiting ultraviolet radiation, and forestalling the degradation of extracellular matrix occasioned by pollution^91^. This further affirms the current usage of biopolymer materials fortified with antioxidants in packaging and active membranes for foods, cosmetics, and pharmaceuticals to reduce lipid peroxidation in such products^10,11^. Furthermore, the hepatoprotective effect of Catechin^13^ corroborates the report of Adewale^92^.

The folkloric applications of *P. guineense* in the treatment of cough, bronchitis, rheumatism and intestinal diseases^8^ could be a function of the reported anti-inflammatory, antispasmodic, antimicrobial abilities of Benzoic acid 4-hydroxy^26^, Lathodoratin^36,37^ and Quinoline-2-(2-pyridinyl)^38^ identified in the spice. Moreover, Adeyi et al*.*^25^ have revealed that a metalloprotease in the venom of the saw-scaled viper, *Echis ocellatus* was inhibited by an ethyl-acetate fraction of the spice containing the bioactive compound, 2-Pentanone, 4-cyclohexylidene-3,3-diethyl-. Thus, this compound may serve as a lead molecule in the synthesis of drugs for combating snake envenoming.

Established antioxidant compounds such as Genkwanin, Apigenin, Thioflavin, Quercetagetin, and others may have been responsible for the reported hepatoprotective activity of *R. beninensis* by Evuen et al*.*^6^. Moreover, the reported estrogenic and anti-inflammatory activity of the spice further affirms the reported aphrodisiac and anti-inflammatory properties of the plant by Ofeimum and Ayinde^12^ and Ofeimum et al*.*^5^ respectively.

## Conclusion

This study has for the first time, revealed the mineral, proximate and bioactive constituents of *R. beninensis* roots. It has also given credence to the folkloric utilization and scientific reports on the three spices evaluated in this study. However, to broaden our horizons on the biological attributes of the spices, it is recommended that the bioactive components are harvested and subjected to further studies to validate their relevance in food preservation, nutraceutical and pharmaceutical production.

## Materials and methods

### Chemicals

All chemicals used in the present study were of analytical grade purchased from Pyrex- IG Scientific Company, Benin City, Nigeria.

### Experimental research and field studies on plants

The study and other experimental procedures employed were as described in the methods. The various spices were collected from the wild which were the source for commercialisation by various marketers of spices. We did not apply research design for plant cultivation. In addition, the study employed basic experiments which include non-human clinical tests, non-animal tests and in vitro tests in natural environmental conditions. The plant collection and use was in accordance with all the relevant guidelines.

### Collection, identification, and pulverization of plant samples

The spices, *Xylopia aethiopica* (Fruits)*, Piper guineense* (seeds), *and Raphiostylis beninensis* (roots) were purchased from a local market in Oghara, Delta State, Nigeria, identified and authenticated at the Herbarium Section of the Department of Plant Biology and Biotechnology, University of Benin, Edo State, Nigeria by Dr. H.A. Akinnibosun. Specimens with voucher numbers, UBHx0348, UBHa0328, and UBHp0262 respectively were deposited in his herbarium. A large quantity of the spices was subjected to room temperature drying at 27.0 ± 2.0 °C for two weeks. Thereafter, the spices were subjected to homogenization using a warring mechanical blender to obtain dried, pulverized plant materials respectively. The pulverized plant materials were then stored in air-tight containers at 4 °C until required for use.

### Mineral analyses of the spices

The concentrations of magnesium, zinc, iron, selenium, copper, calcium, manganese, molybdenum, potassium, and sodium in the spice samples were ascertained by using the Atomic Absorption spectrophotometer (SP9, Pychicham, UK) according to the method described by the Association of Official Analytical Chemists^93^.

### Phytochemical analysis of the spice

The tannin content of the samples was determined by Folin Denis colorimetric method^94^. Alkaloids were quantitatively determined according to the method of Harborne^95^. Flavonoids were determined using the method described by Harbone^96^. Quantitative determination of Oxalate was carried out using the method reported by Ejikeme et al.^97^. Phytates were determined through phytic acid determination using the procedure described by Akaneme et al.^98^. The determination of saponins was done following the method of Obadoni and Ochuko^99^ and total phenol in the plant extracts was determined according to the method of the Association of Official and Analytical Chemists^93^*.*

### Proximate nutrient analysis of the spices

The crude fibre, crude protein, fat, moisture, and total ash contents of samples were analyzed using standard protocols^93,100–103^. The total carbohydrate was determined by difference; the sum of the percentage moisture, ash, crude lipid, crude protein and crude fibre was subtracted from 100 [104].

### Gas chromatography–mass spectrometry analysis (GC–MS)

The methanol extracts of the three spices were subjected to the Gas Chromatography and Mass Spectrometry (GC–MS) analysis to reveal their bioactive components. Three microliters **(**3 uL) of each of the sample extracts were injected into the GC column for analysis. The GC (Agilent 6890 N) and MS (5975B MSD) were equipped with a DB-5 ms capillary column (30 m × 0.25 mm; film thickness 0.25 µm). The initial temperature was set at 40 °C which increased to 150 °C at the rate of 10 °C/min. The temperature was again increased to 230 °C at the rate of 5 °C/min. The process continued until a value of 280 °C temperature was attained at the rate of 20 °C/min which and held for 8 min. The injector port temperature remained constant at 280 °C and the detector temperature was at 250 °C. Helium was used as the carrier gas with a flow rate of 1 mL/min. The split ratio and ionization voltage were 110:1 and 70 eV respectively.

To identify the unknown chemical components present in the samples, their individual mass spectral peak value was compared with the database of the National Institute of Science and Technology, 2014. Thereafter, the chemicals were identified by comparing the unknown peak value and chromatogram from GC–MS against the known chromatogram peak value from the National Institute of Standards and Technology (NIST) Library database. Subsequently, the details about their molecular formula, molecular weight, retention time and percentage content were also obtained.

### Data analysis

Data obtained from this study were subjected to analysis of variance (ANOVA) using the statistical package (SPSS 21.0). Results were expressed as Mean ± S.E.M. of three replicate determinations. Mean values of various groups were significantly compared by Tukey’s Multiple Range Test and a probability of *p* < 0.05 was considered significant.

## Data Availability

The datasets used and/or analyzed during the current study are available from the corresponding author on reasonable request.
